# The Social Cost of Perseverative Cognition: Impairing Interpersonal Engagement Propensity Through Cognitive and Autonomic Dysregulation

**DOI:** 10.1111/psyp.70379

**Published:** 2026-08-17

**Authors:** Martina Fusaro, Chiara Fini, Giovanna Cuomo, Seyma Kaya, Cristina Ottaviani, Vanessa Era

**Affiliations:** ^1^ Department of Psychology Sapienza University Rome Italy; ^2^ LIFE Institute for Research and Health Care‐Santa Lucia IRCCS Rome Italy; ^3^ Department of Psychology and Health Sciences Pegaso Telematic University Naples Italy; ^4^ Department of Psychology Philipps University Marburg Marburg Germany

**Keywords:** heart rate variability, inclusion of other in the self, interpersonal closeness, interpersonal engagement, perseverative cognition

## Abstract

Perseverative cognition, the repetitive, sustained activation of cognitive representations about past stressors or anticipated threats, is known to impair attention and disrupt emotional and physiological regulation; yet, its interpersonal consequences remain poorly understood. This study tested whether a brief induction of perseverative cognition reduces interpersonal engagement propensity, defined as the willingness to initiate social interactions. In a within‐subjects design, participants completed two counterbalanced sessions: (i) a perseverative cognition induction, involving the recall and description of a personally salient, recurrent, and emotionally distressing event; and (ii) a control condition, involving the description of the plot of a dramatic but non‐self‐referential film or book. Cognitive, emotional, and physiological responses were assessed using self‐reported measures of thought distraction, negative affect, heart rate (HR), and vagally mediated HR variability (HRV), respectively. Interpersonal engagement propensity was evaluated through perceived self‐other overlap (Inclusion of the Other in the Self, IOS), preferred interpersonal closeness, and automatic imitation. Compared with the control condition, the perseverative cognition induction elicited higher distraction, greater negative affect, elevated HR, and reduced HRV. Greater thought distraction and lower HRV were associated with reduced preferred interpersonal closeness, indicating that cognitive interference and diminished parasympathetic regulation may both contribute to reduced willingness to engage with others following perseverative cognition. These results suggest that perseverative cognition can undermine social engagement in healthy individuals, highlighting its potential role as a transdiagnostic mechanism contributing to social withdrawal.

## Impact Statement

Perseverative cognition is known to impair emotional and physiological regulation, yet its interpersonal consequences remain poorly understood. This study demonstrates that even brief episodes of perseverative thinking reduce individuals' propensity to engage particularly among individuals showing greater cognitive distraction and lower vagally mediated heart rate variability, highlighting a potential pathway linking internal distress to social withdrawal.

## 1 Introduction

Perseverative cognition refers to the repetitive and sustained activation of thoughts related to past or anticipated stressors. It encompasses rumination, which is past‐oriented, and worry, which is future‐oriented and represents a transdiagnostic process implicated in several emotional disorders, including generalized anxiety disorder, major depression, post‐traumatic stress disorder, and social anxiety disorder (Ehring and Watkins 2008). According to the Perseverative Cognition Hypothesis (Brosschot et al. 2006), the prolonged mental representation of negative content elicits physiological stress activation, even in the absence of external threats. This is reflected in heightened sympathetic activation (e.g., elevated heart rate, HR) and reduced parasympathetic activity, indexed by lower vagally mediated HR variability (HRV), which is widely recognized as a marker of self‐regulation and emotional flexibility (Thayer and Lane 2009; Beauchaine and Thayer 2015).

While research has extensively examined the intrapersonal effects of perseverative cognition, including impaired attentional resources (Fox et al. 2015; DeGennaro et al. 2025), emotional dysregulation (Brosschot et al. 2006), and altered autonomic activity (Ottaviani et al. 2016), its interpersonal consequences remain underexplored. This gap is notable, as social dysfunction is a core component of many clinical disorders involving perseverative cognition. For example, individuals with depression or social anxiety commonly exhibit reduced motivation for social interaction, heightened sensitivity to social rejection, and diminished perceived social support (Segrin 2000; APA 2013). It is plausible that perseverative thinking contributes to these impairments by disrupting attentional, emotional, and physiological systems essential for adaptive social functioning.

Interpersonal space regulation represents a likely domain where such impairments may emerge, including both interpersonal closeness, defined as the preferred spatial distance maintained during social interactions (Hall 1966; Hayduk 1978) and self‐other overlap (Aron et al. 1992), defined as perceived emotional connectedness. For instance, studies have shown that individuals with social anxiety and depressive traits tend to prefer greater interpersonal distance, especially when interacting with strangers (Wieser et al. 2010). Similarly, depressed individuals often experience reduced interpersonal connectedness, regardless of actual social contact (Lüönd et al. 2022). For example, Lüönd et al. (2022) found that both depression and a history of childhood maltreatment predicted increased physical distancing, suggesting that early adverse experiences and depressive symptomatology may shape implicit social avoidance tendencies. Interpersonal closeness and self‐other overlap represent related yet distinct aspects of social connectedness. Both interpersonal closeness and self‐other overlap reflect affiliative motivation; however, self‐other overlap is more sensitive to emotional states and is more related to feelings of “being connected” (Zhang and Hommel 2022), whereas interpersonal closeness captures a readiness‐to‐interact dimension that is more linked to approach–avoidance motivation (Kennedy et al. 2009). In addition to interpersonal closeness and self‐other overlap, automatic imitation offers a non‐verbal, implicit indicator of social affiliation. Defined as the spontaneous copying of others' gestures or movements, automatic imitation fosters interpersonal relationships and is modulated by affective and motivational states (Lakin et al. 2003; Leighton et al. 2010; Genschow and Schindler 2016).

Existing evidence suggests that perseverative cognition may compromise interpersonal engagement propensity, defined here as the motivation to approach and engage with others, by dysregulating multiple interdependent systems. At the physiological level, it alters autonomic balance by enhancing sympathetic activation and reducing vagal tone, affecting emotion regulation (Brosschot et al. 2006). Moreover, elevated HR has been linked to heightened interoceptive threat sensitivity, wherein individuals may misinterpret internal bodily cues (e.g., cardiac acceleration) as indicative of external danger or personal vulnerability (Critchley et al. 2004; Domschke et al. 2010; Paulus and Stein 2006). Such misinterpretations are thought to amplify hypervigilance and foster exaggerated perceptions of threat in the social environment (Dunn et al. 2007), possibly diminishing one's propensity to engage in interpersonal interactions. At the cognitive level, perseverative thoughts hinder attentional disengagement from self‐referential content (Koster et al. 2011), possibly limiting an individual's ability to process and respond adaptively to socially relevant stimuli. At the affective level, they are associated with intensified negative affect (Nolen‐Hoeksema et al. 2008), which has been shown to bias motivational systems toward avoidance and reduce sensitivity to affiliative signals (Keltner and Kring 1998). These affective states may undermine the emotional attunement necessary for reciprocal engagement (Fini et al. 2023). Collectively, these physiological, cognitive, and affective changes are hypothesized to reduce interpersonal engagement propensity.

To the best of our knowledge, however, no prior study has experimentally manipulated perseverative thinking in healthy participants to test its role in modulating interpersonal engagement propensity. The present study aimed to address this gap by examining whether, and through which mechanisms, perseverative cognition impairs interpersonal engagement propensity. In a within‐subjects design across two counterbalanced sessions, participants were asked to recall and describe either a personally salient, recurrent, and emotionally distressing event (perseverative cognition condition) or the plot of a recently viewed dramatic film, series, or book unrelated to their personal experience (control condition). To assess the efficacy of the induction, we measured subjective thought‐related distraction, negative affect, HR, and vagally mediated HRV, capturing the cognitive, emotional, and physiological impact of the tasks. Interpersonal engagement propensity was subsequently assessed using three distinct indicators: perceived self‐other overlap, preferred interpersonal closeness, and automatic imitation. We hypothesized that, relative to the control condition: (i) the perseverative cognition induction would evoke greater thought‐related distraction, heightened negative affect, elevated HR, and reduced HRV; and that (ii) these responses would be associated with reduced interpersonal engagement propensity, evidenced by lower interpersonal closeness, diminished self‐other overlap, and attenuated automatic imitation. Clarifying how perseverative thinking undermines interpersonal functioning may refine theoretical accounts of social cognition and inform clinical interventions for improving social connection in internalizing psychopathologies.

## 2 Materials and Methods

### 2.1 Participants

A priori power analysis was conducted using MorePower software. Based on a medium‐to‐large effect size (*η* ^2^ = 0.15) (Soliman et al. 2015), an alpha level of 0.05, and a desired power of 0.80 for a within‐subjects design with one two‐level factor, a sample size of 48 participants was estimated. Forty‐nine healthy volunteers (19 males and 30 females) aged 18–40 years (*M* = 24.55, SD = 3.16) participated in this study. Participants were recruited based on the following inclusion criteria: absence of any cardiac or cardiovascular diseases, absence of any neurological or psychiatric disorders, absence of alcohol or substance abuse, and a body mass index (BMI) < 30 kg/m^2^. Participants provided informed consent after reviewing an information sheet. The study was approved by the ethical committee of Santa Lucia Foundation (prot n CE/2023_016).

To further characterize the sample, participants completed the Rumination Response Scale (RRS; Treynor et al. 2003), a 10‐item self‐report scale assessing the dispositional tendency to engage in depressive ruminative thoughts; the Penn State Worry Questionnaire (PSWQ; Meyer et al. 1990), a 16‐item scale assessing individual differences in the tendency to worry, independently of anxiety and depression; the State–Trait Anxiety Inventory (STAI; Spielberger et al. 1983), a 20‐item scale measuring both state and trait anxiety; the UCLA Loneliness Scale (Version 3; Russell 1996), a 20‐item scale assessing subjective feelings of loneliness and social isolation; and the Transgression‐Related Interpersonal Motivations Inventory (TRIM‐18; McCullough et al. 2013), an 18‐item measure designed to assess interpersonal motivations following a harmful transgression. The TRIM‐18 includes three subscales: Avoidance (e.g., “I live as if he or she doesn't exist”), Revenge (e.g., “I'll make him/her pay”), and Forgiveness (e.g., “Even though his/her actions hurt me, I have goodwill for him/her”). A description of the questionnaires scores is provided as Table S1.

### 2.2 Physiological Measurements

HR and HRV were continuously recorded using a Biopac System with a Lead I electrocardiogram (ECG) configuration. Three electrodes were placed on the participant: two recording electrodes and a ground electrode. The negative electrode was positioned on the right upper chest, the positive electrode on the left upper chest, and the ground electrode was placed between the last rib and the upper abdomen. HR and HRV were assessed at baseline and during the experimental conditions. Baseline measures were collected at the beginning of the experimental session, during a five‐minute resting period while participants viewed a neutral video of a flowing stream (vanilla baseline; Jennings et al. 1992).

### 2.3 Experimental Procedure

**FIGURE 1:**
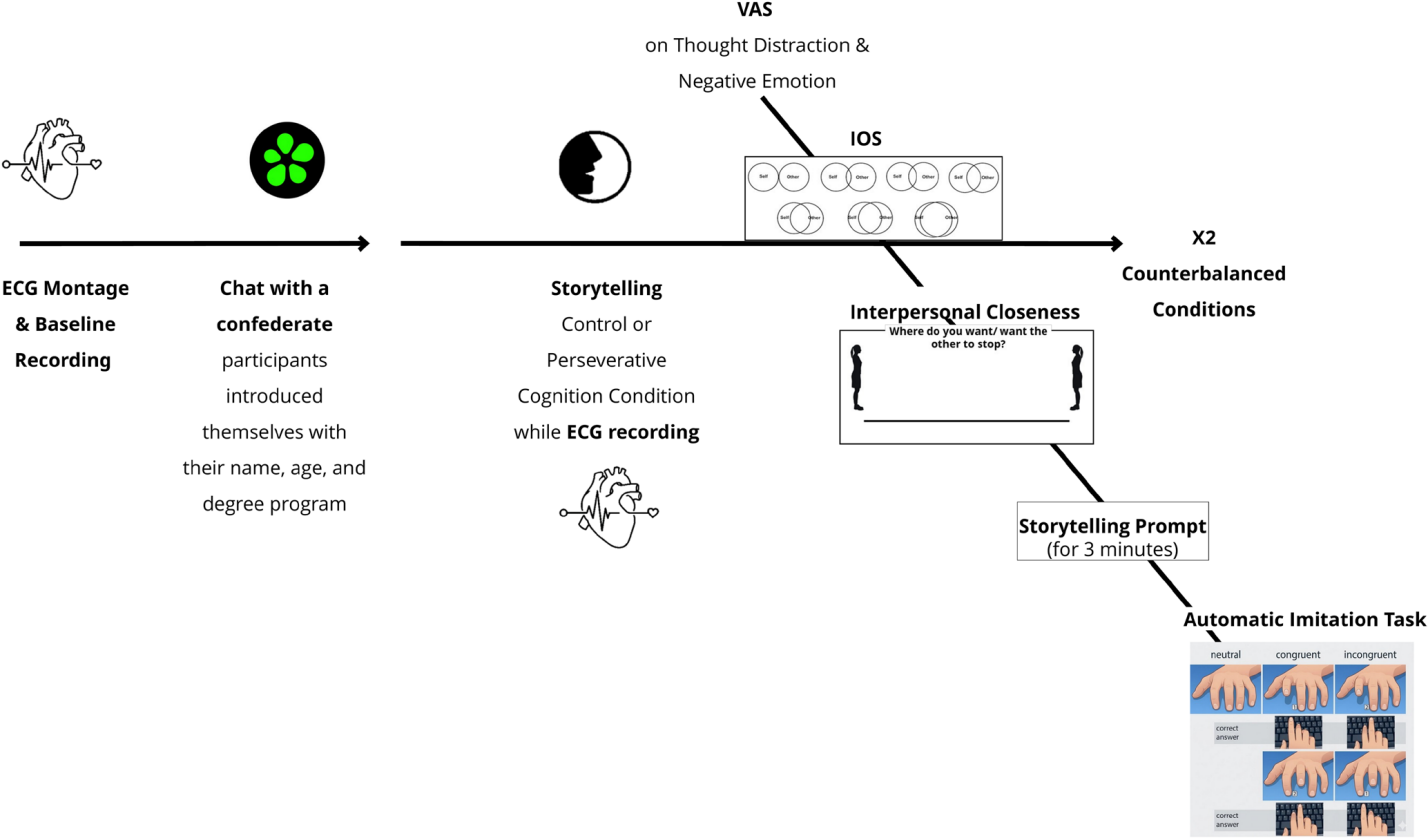
Graphical visualization of the experimental timeline.

Upon arrival, participants signed an informed consent form. Electrodes were then attached for ECG assessment, and baseline physiological measurements were recorded. Following this, participants engaged in a 2‐min online chat session via ICQ (an instant messaging software; see Fini et al. 2025 for a similar procedure). The interaction was conducted exclusively via keyboard, with both participants and the confederate communicating by typing messages. As part of the experimental cover story, participants were led to believe they would be chatting with another gender matched participant who had also completed the same preliminary steps and would be performing a series of tasks with them. Participants were told: “You will be asked to chat for two minutes with a person who is participating like you in this experiment. In the chat, you will introduce yourself, then perform a series of tasks together with this person.” The conversation partner was actually a member of the research team who followed a standardized script (e.g., providing the same information about name, age, and degree program and responding using predefined messages), ensuring consistent interactions across participants. After each chat a storytelling session started, where the participants were asked to recall and narrate an event in two counterbalanced conditions, one involving the induction of perseverative cognition and the other serving as a control condition. Then the experimental session would start, involving several measures described below; before the last task, participants would be prompted to recall the content of the storytelling session. Each participant completed two experimental sessions, separated by 1 week, in a counterbalanced order: one session involving the induction of perseverative cognition and the other the control condition. To control for the effects of speaking aloud itself on the cardiovascular and autonomic nervous systems (e.g., Lynch et al. 1981), participants completed the same verbal narration procedure in both sessions, while the experimenter provided only standardized prompts to facilitate elaboration. The experimenter's role was limited to maintaining participants' engagement in the narration and was identical across both conditions. Both conditions lasted 3 min, thereby ensuring equivalent task duration and comparable motor demands across conditions. See Figure 1 for a graphical description of the experimental procedure.

#### 2.3.1 Storytelling

##### 2.3.1.1 Induction of Perseverative Cognition

In the perseverative cognition induction condition, participants were instructed to recall and describe a past or future event that they often thought about unintentionally and caused feelings of sadness, anxiety, anger or worry. This is a widely replicated procedure that has been shown to be effective in eliciting perseverative cognition in both clinical and non‐clinical samples (e.g., Ottaviani et al. 2013; Ottaviani 2018; Era et al. 2021). Participants were encouraged to vividly recall and articulate the details of the event or scenario, including the associated thoughts, emotions, and physical sensations they experienced. To facilitate this process, participants narrated the event aloud while the experimenter listened and provided only standardized verbal prompts to encourage further elaboration. For instance, the experimenter could ask follow‐up questions such as, “How did you feel at that moment?” or “Can you describe what thoughts came to your mind during the event?” No additional feedback, interpretation, or conversation beyond these standardized prompts was provided. This approach was designed to help participants to explore deeper into their emotional experiences, ensuring the effective induction of perseverative thinking and elicitation of the corresponding emotional states.

##### 2.3.1.2 Control Condition

In the control condition, participants were asked to narrate the storyline of a movie or TV series they had recently whatched, or a book they had recently read, that contained dramatic content that was unrelated to their personal experiences. This condition was specifically designed to maintain engagement with negatively valenced material, while minimizing the repetitive, unwanted and intrusive self‐referential processing that characterizes perseverative cognition. Participants narrated the storyline aloud while the experimenter listened and provided only standardized prompts (e.g., “What was the main story about?” or “What were the most important events or characters in the story?”) to encourage elaboration. No additional feedback or conversation beyond these standardized prompts was provided. This procedure aimed to ensure that both experimental conditions were comparable in terms of emotional valence and arousal, while differing in terms of the other key characteristics of perseverative cognition, namely personal relevance, repetitiveness, intrusiveness and uncontrollability (e.g., Ehring and Watkins 2008). Both conditions were standardized to last 3 min to ensure uniformity in the duration of exposure to the experimental and control tasks.

##### 2.3.1.3 Prompt

Prior to the final experimental task, participants were instructed to spend 3 min recalling the content of the storytelling session.

### 2.4 Visual Analogue Scale (VAS)

Following the induction of perseverative cognition and control conditions, a Visual Analogue Scale (VAS) was used to measure participants' level of distraction and negative emotional states. Two questions were asked: “How much are you currently distracted by your thoughts?” and “Are you currently experiencing negative emotions?” Responses ranged from 0 (not at all) to 100 (extremely).

### 2.5 Behavioral Tasks

#### 2.5.1 Inclusion of the Other in the Self (IOS)

After the VAS questions, the IOS scale (Aron et al. 1992), a single‐item pictorial measure designed to evaluate perceived self‐other overlap between two individuals, was administered. The IOS scale presents participants with six pairs of circles ranging from completely separate to nearly fully overlapping, each representing varying degrees of closeness between “Self” and “Other”. Hence, the IOS Ratings varied from 1 to 6. Participants were asked to select the pair of circles that best represented their perceived self‐other overlap with the person they interacted with during the chat session at the start of the experiment.

#### 2.5.2 Interpersonal Closeness

After the IOS, Interpersonal Closeness preferences were assessed using a task adapted from Lisi et al. (2021) to measure the spatial comfort zone participants preferred when visualizing their interaction partner in front of them. Participants were presented with a digital representation of themselves and their chat partner, represented by gender‐matched silhouettes placed on a horizontal scale. The virtual avatars were realized using MakeHuman Community 1.2.0 (http://www.makehumancommunity.org); the pictures of the avatars were generated using Unity v.2019.4.15f1 (https://unity.com). The silhouette representing the participant was positioned on the far‐left end of the scale, and the silhouette representing their chat partner was positioned further to the right. Participants were asked to indicate both passive and active interpersonal closeness preferences. For the passive closeness condition, they received the following instructions: “Imagine that you are the figure positioned at the left end of the line and that you are unable to move or turn. Now, envision the person you just interacted with, represented on the right end of the line, walking toward you. Please indicate the point at which you would feel comfortable having this person stop, by clicking on the horizontal line. Then, press ‘Next’ to continue.” In the active closeness condition, participants were instructed as follows: “In this scenario, imagine that you are moving toward the other person along the same horizontal line. Indicate the point at which you would choose to stop while still feeling comfortable. Click on the line to record your response, then press ‘Next’ to proceed.” This task was conducted to assess the participants' inclination toward interpersonal closeness or avoidance after the experimental conditions. The response values were recorded on a scale from 0 (no movement of the avatar) to 100 (complete overlap with the partner silhouette). Higher values indicated greater interpersonal closeness, corresponding to a reduced preferred interpersonal distance from the interaction partner.

#### 2.5.3 Automatic Imitation Task

Finally, the automatic imitation task, developed by Brass et al. (2001), was used to evaluate participants' tendency to unconsciously imitate the actions of another individual (experimental script made available by Westfal et al. 2025). Participants viewed a series of images of a hand performing finger movements (index or middle finger) on a computer screen. Simultaneously, they were instructed to respond to numerical cues (e.g., “1” or “2”) by lifting their index or middle finger as quickly as possible. Three types of trials were included: (1) Congruent trials in which the observed hand movement matched the required finger movement (e.g., the image showed an index finger lifting, and the participant was cued to lift their index finger); (2) Incongruent trials in which the observed hand movement did not match the required finger movement (e.g., the image showed a middle finger lifting, but the participant was cued to lift their index finger); and (3) Neutral trials in which only the numerical cue was presented, participants had to lift their finger, but no hand movement was observed. Each trial began with a 500 ms presentation of a neutral hand image. The second image displays a lifting movement for 2000 milliseconds. Participants performed a practice condition involving 12 stimuli, comprising 4 congruent, 4 incongruent, and 4 neutral stimuli. Following this stage, participants were instructed to complete the task consisting of a total of 150 separate trials, with 50 trials each for neutral, incongruent, and congruent conditions. The presentation order of these trials was randomized. Reaction times (RTs) were recorded for all trial types. RTs were defined as the duration from target onset to the moment participants lifted their finger from the ‘g’ or ‘h’ key. An index of facilitation (RTs in congruent minus neutral trials), one of interference (RTs in incongruent minus neutral trials) and one of congruence (RTs in congruent minus incongruent trials) were computed. Because responses are typically faster in congruent trials, facilitation values are expected to be negative, with more negative values indicating stronger facilitation effects.

### 2.6 Statistical Analyses

All analyses were conducted using R version 4.5.2 and are available on OSF (https://osf.io/udxhs/overview?view_only=3bf64b0db8824a91bee383b679188edb).

#### 2.6.1 Computation of HR and HRV

ECG signals were processed using Kubios HRV Premium (Tarvainen et al. 2014). RR interval series were visually inspected, and artifacts and ectopic beats were identified and corrected using Kubios' automatic artifact‐correction algorithm, which replaces affected RR intervals by interpolation prior to HRV estimation. Participants whose ECG recordings remained of insufficient quality after preprocessing were excluded from the cardiac analyses. Across all retained ECG recordings, the proportion of corrected RR intervals was generally low (median = 0.66%, IQR = 0.00%–2.17%; mean = 1.52%, SD = 2.40%; range = 0.00%–23.53%). Overall, 39.3% of recordings required no beat correction, whereas only 7.3% required correction of more than 5% of RR intervals and 1.3% exceeded 10% (Table S2).

The calculation of vagally mediated HRV was performed by assessing the root mean square of the differences between consecutive RR intervals (RMSSD, measured in milliseconds). Due to bad signal quality, the recording of HR and HRV measures was restricted to 39 participants.

#### 2.6.2 Effectiveness of Perseverative Cognition Induction Measured as Subjective Reports and Physiological Responses

To assess the effectiveness of the perseverative cognition induction, paired‐sample Wilcoxon signed‐rank tests compared participants' subjective reports from the VAS across the experimental and control conditions, using the rstatix package in R (Kassambara 2025). The two key variables were: (1) the extent of Thought Distraction (“How much are you currently distracted by your thoughts?”) and (2) the experienced Negative Emotions (“Are you currently experiencing negative emotions?”).

To evaluate the effectiveness of the perseverative cognition induction in eliciting a physiological stress response, typically associated with perseverative thinking (Ottaviani et al. 2016), HR and HRV were analyzed and compared to baseline values. Specifically, the three‐minute storytelling phases were divided into three consecutive 1‐min segments to assess temporal fluctuations in physiological responses. This segmentation allowed for a detailed examination of dynamic changes in HR and HRV throughout the task, enabling comparisons between baseline measurements and those recorded during both the perseverative cognition induction and control conditions. Since the Shapiro–Wilk tests indicated some deviations from normality in certain conditions, we used a 2 × 4 repeated‐measures parametric ANOVA via the Artool package (Wobbrock et al. 2011). The within‐subject factors were Condition (perseverative cognition, control) and Time (three consecutive 1‐min segments during the task: time 1, time 2, time 3, plus a pre‐task baseline). This design allowed us to assess both the overall effect of thought manipulation and the temporal evolution of physiological responses across the task phases. Bonferroni corrections were applied to account for multiple comparisons.

#### 2.6.3 Impact of Perseverative Cognition Induction, Subjective Reports and Physiological Responses on Inclusion of the Other in the Self (IOS)

Data from the IOS scale were analyzed using cumulative link mixed models (CLMM), implemented via the ordinal package in R (Christensen 2023) and estimated using the Laplace approximation to appropriately model the ordinal nature of the dependent variable. As there were no repeated trials within each experimental condition, participants were included as random intercepts without random slopes. To evaluate the robustness of the mixed‐effects results given the relatively modest sample size, participant‐level cluster bootstrap (based on 1000 permutations) analyses were additionally performed to obtain bootstrap confidence intervals while preserving the within‐participant dependency structure. The experimental Condition (perseverative cognition vs. control) was entered as a fixed effect. To examine the influence of subjective experiences, including the extent of Thought Distraction, Negative Emotion, and Physiological Responses (HR and vagally mediated HRV in the first minute of the storytelling task), on perceived IOS, each predictor was centered and entered as a covariate in separate models. Fixed effects were evaluated using Wald z‐tests from the model summary. To interpret potential interactions between the categorical variable (Condition) and continuous predictors, estimated marginal trends were computed using the emtrends function from the emmeans package (Lenth and Piaskowski 2025). This approach allowed us to test whether the slope of the continuous predictor was significantly different from zero within each condition and whether these slopes differed significantly between conditions. Bonferroni corrections were applied to account for multiple comparisons.

#### 2.6.4 Impact of Perseverative Cognition Induction, Subjective Reports and Physiological Responses on Interpersonal Closeness

Interpersonal closeness preferences were analyzed using generalized linear mixed models (GLMMs) with a beta distribution and logit link, implemented via the glmmTMB package in R (Brooks et al. 2017) and estimated using maximum likelihood. Because distance ratings were bounded between 0 and 100, values were first rescaled into the (0,1). As each experimental Condition (perseverative cognition vs. control) was presented only once and without repeated trials, participants were modeled with random intercepts and no random slopes. To assess the robustness of the mixed‐effects models, parametric bootstrap likelihood‐ratio tests based on 1000 simulations were performed on the final models. The fixed effects included the experimental Condition (perseverative cognition vs. control) and the Type of Closeness (active vs. passive). To examine the influence of subjective experiences, such as the extent of Thought Distraction, Negative Emotion, and Physiological Responses (HR and vagally mediated HRV during the first minute of the storytelling task), each continuous predictor was centered and entered in a separate model as a covariate interacting with Condition and Distance Type. Slope estimates for continuous predictors within each level of the experimental condition were computed using the emtrends function (Lenth and Piaskowski 2025) as for the analyses on IOS ratings. Bonferroni corrections were applied to account for multiple comparisons.

#### 2.6.5 Impact of Perseverative Cognition Induction, Subjective Reports, and Physiological Responses on Automatic Imitation

Automatic imitation was assessed using three indices computed from RTs: Facilitation (Congruent—Neutral), Interference (Incongruent—Neutral) and Congruence (Incongruent—Congruent), capturing the extent to which observed actions respectively facilitated or interfered with participants' motor responses. Before creating the indexes, the dataset was cleaned of inaccurate trials (3.5%) and RTs that were above or below 2.5 SD (2%), which were considered outliers. Two subjects were excluded from the analyses: (i) one participant presenting only one accurate Incongruent trial after induction of the Perseverative Cognition (ii) one participant whose mean reaction times were more than 2.5 standard deviations above or below the group mean. All the other subjects performed above the 50% accuracy in each experimental Condition (Perseverative Cognition vs. Control) and Trial (Congruent, Incongruent, Neutral). Each index was analyzed separately using linear mixed‐effects models (LMMs) implemented with the lme4 (Kuznetsova et al. 2017) package in R. Experimental Condition (Perseverative Cognition vs. Control) was included as a fixed factor, and participants were modeled with both random intercepts and random slopes for Condition, given the presence of multiple trials per subject.

To examine the role of subjective experiences in modulating imitation behavior, separate models were fitted including Thought Distraction and Negative Emotion as continuous predictors, and Condition as a fixed factor. Physiological responses, HR and vagally mediated HRV recorded during the first minute of the storytelling task, were also inserted as continuous predictors in separate models using the same structure. All continuous predictors were centered before the analyses. Type III ANOVA was used to determine the significance of fixed effects. For statistically significant fixed effects, robustness was further assessed using parametric bootstrap likelihood‐ratio tests.

## 3 Results

### 3.1 Effectiveness of Perseverative Cognition Induction Measured as Subjective Reports and Physiological Responses

#### 3.1.1 Thought Distraction

**FIGURE 2:**
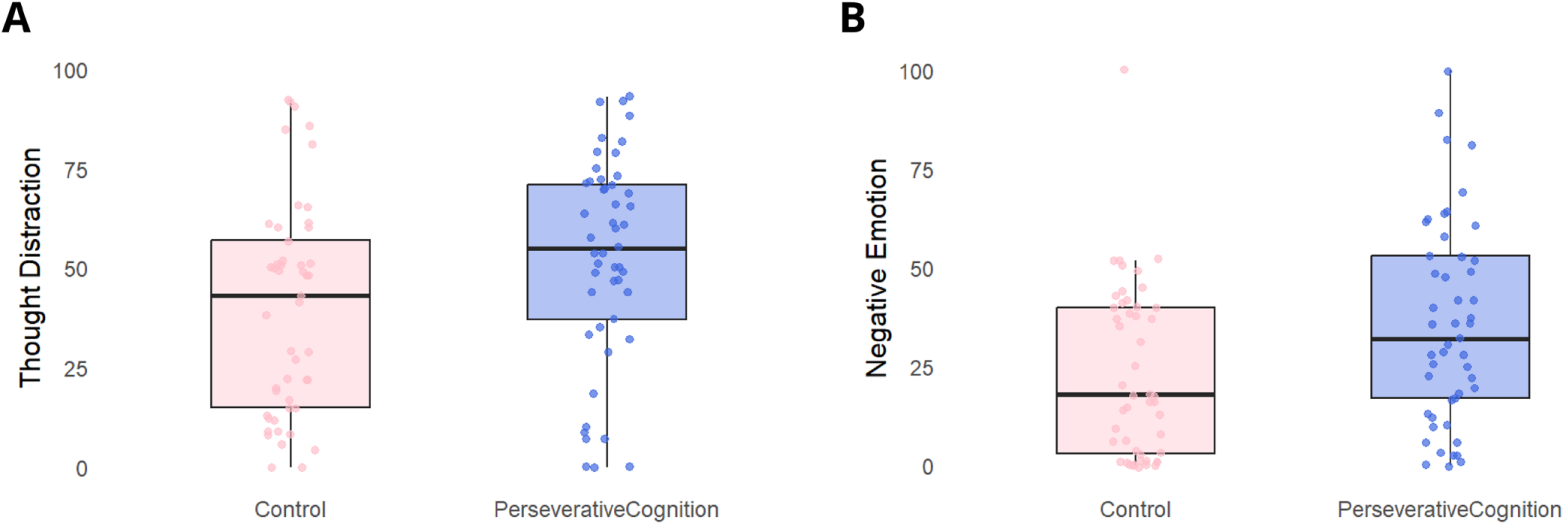
Panel A: Paired‐sample Wilcoxon signed‐rank test showed that participants reported significantly higher levels of thought distraction in the perseverative cognition condition compared to the control condition (*p* = 0.007). Panel B: Participants also reported significantly higher levels of negative emotion in the perseverative cognition induction condition compared to the control condition (*p* = 0.002). The plots display individual participants' mean scores (dots) in the perseverative cognition and control conditions. The boxplots indicate the median and interquartile range.

Paired‐sample Wilcoxon signed‐rank test showed that the extent of Thought Distraction was significantly higher in the perseverative cognition induction condition (*M* = 52.61, SD = 26.09) compared to the control condition (*M* = 39.74, SD = 26.68), *V*(48) = 850, *p* = 0.007 (Figure 2, panel A).

#### 3.1.2 Negative Emotion

Similarly, the experienced Negative Emotions were significantly higher in the perseverative cognition induction condition (*M* = 35.67, SD = 25.42) compared to the control condition (*M* = 22.98, SD = 21.67), *V*(48) = 865, *p* = 0.002 (Figure 2, panel B).

#### 3.1.3 HR

**FIGURE 3:**
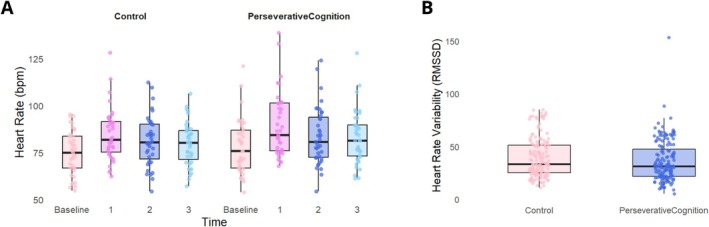
Panel A: The plots display heart rate (HR, bpm) measured across three time points during the task (1, 2, and 3) and a pre‐task baseline, separately for the perseverative cognition and control conditions. Overall, HR was higher in the perseverative cognition condition compared to the control condition, reflecting greater physiological activation after perseverative cognition induction. Across both conditions, HR was significantly elevated during the task compared to baseline, with the highest values observed at the beginning of the storytelling phase (time 1). This initial peak was followed by a progressive decrease over time, suggesting a habituation effect as the task unfolded. Each dot represents a participant's mean HR at each time point. Boxplots indicate medians and interquartile ranges. Panel B: The plot displays the main effect of condition in HRV (RMSSD), showing higher levels of HRV in the control condition compared to the perseverative cognition induction (*p* < 0.01). Each dot represents a participant's mean HRV and boxplots indicate medians and interquartile ranges.

The repeated‐measures aligned transform rank (ART) ANOVA on HR revealed a significant main effect of Condition (*F*(1,38) = 14.84, *p* < 0.001), indicating that HR was higher in the perseverative cognition condition (*M* = 83.61; SD = 15.62) compared to the control condition (*M* = 80.11; SD = 12.87). There was also a significant main effect of Time (*F*(3,114) = 32.76, *p* < 0.0001), but no significant interaction between Time and Condition (*p* = 0.65). Bonferroni‐corrected post hoc tests showed that HR was significantly higher at all three task time points (Time 1, 2, and 3) compared to the baseline (all *ps* < 0.001). Moreover, HR was significantly higher at the beginning of the storytelling phase (Time 1; *M* = 86.95, SD = 15.56) compared to time 2 (*M* = 82,45, SD = 14.16), time 3 (*M* = 81.43, SD = 12.94) and baseline (*M* = 76.63, SD = 13.13; all *ps* < 0.001), but not at time 2 compared to time 3 (*p* = 1.00) indicating an initial higher HR that gradually decreased over the course of the task (Figure 3, Panel A).

#### 3.1.4 HRV

The same repeated‐measures ART ANOVA conducted on HRV values did not reveal any significant main effect of Time or interaction between Time and Condition (all *ps* > 0.14), but showed a main effect of Condition (*F*(1,38) = 8.01, *p* < 0.01). HRV was higher in the control condition (*M* = 39.46; SD = 18.77) compared to the perseverative cognition one (*M* = 36.23; SD = 19.50) (Figure 3, Panel B).

### 3.2 Impact of Perseverative Cognition Induction, Subjective Reports and Physiological Responses on Inclusion of the Other in the Self (IOS)

#### 3.2.1 Thought Distraction

The model including Thought Distraction as a continuous predictor, Condition as a fixed factor, and IOS ratings as the dependent variable revealed no significant effects (all *ps* > 0.11). Participant‐level cluster bootstrap analyses based on 1000 resamples likewise did not provide evidence for the effects of Condition (pboot = 0.086, 95% bootstrap CI [−2.732, 0.108]), Thought Distraction (pboot = 0.532, 95% bootstrap CI [−0.044, 0.022]), or their interaction (pboot = 0.376, 95% bootstrap CI [−0.031, 0.108]).

#### 3.2.2 Negative Emotion

The model including Negative Emotion as a continuous predictor, Condition as a fixed factor, and IOS ratings as the dependent variable identified significant main effects of Condition (Estimate = −0.625, *z* = −254.29, *p* < 0.001) and Negative Emotion (Estimate = −0.019, *z* = −8.12, *p* < 0.001), as well as a significant Condition × Negative Emotion interaction (Estimate = 0.018, *z* = 7.61, *p* < 0.001). However, participant‐level cluster bootstrap analyses did not support the robustness of any of these effects: Condition (pboot = 0.170, 95% bootstrap CI [−1.765, 0.313]), Negative Emotion (pboot = 0.308, 95% bootstrap CI [−0.062, 0.021]), or the Condition × Negative Emotion interaction (pboot = 0.400, 95% bootstrap CI [−0.030, 0.078]).

#### 3.2.3 HR

The model including HR as a continuous predictor identified significant main effects of Condition (Estimate = −0.398, *z* = −182.23, *p* < 0.001) and HR (Estimate = −0.035, *z* = −16.22, *p* < 0.001), as well as a significant Condition × HR interaction (Estimate = −0.006, *z* = −2.73, *p* = 0.006). However, participant‐level cluster bootstrap analyses did not support the robustness of these effects: Condition (pboot = 0.468, 95% bootstrap CI [−1.682, 0.623]), HR (pboot = 0.374, 95% bootstrap CI [−0.202, 0.042]), or the Condition × HR interaction (pboot = 0.833, 95% bootstrap CI [−0.096, 0.090]).

#### 3.2.4 HRV

The model including vagally mediated HRV as a continuous predictor did not reveal any significant main effects or interactions (all *ps* > 0.27). Participant‐level cluster bootstrap analyses were consistent with these null findings, providing no evidence for the effects of Condition (pboot = 0.236, 95% bootstrap CI [−1.761, 0.376]), HRV (pboot = 0.909, 95% bootstrap CI [−0.081, 0.078]), or the Condition × HRV interaction (pboot = 0.879, 95% bootstrap CI [−0.064, 0.115]).

### 3.3 Impact of Perseverative Cognition Induction, Subjective Reports and Physiological Responses on Interpersonal Closeness

#### 3.3.1 Thought Distraction

**FIGURE 4:**
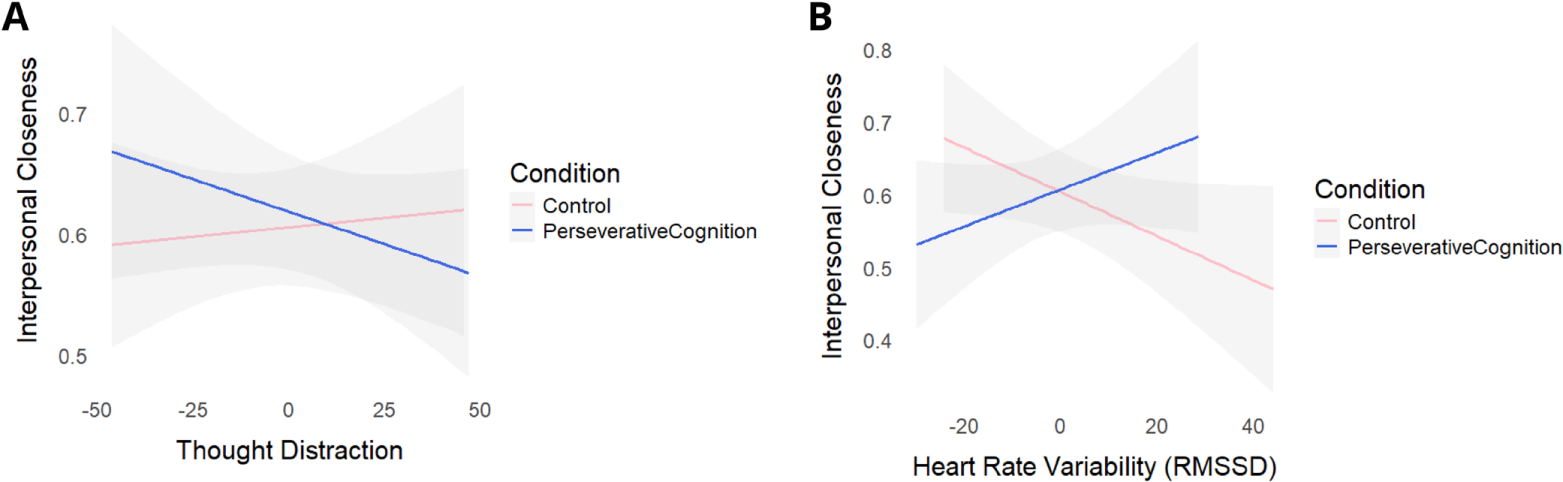
Panel A: The interaction between Condition and Thought Distraction shows that interpersonal closeness decreased as distraction from thoughts increased in the perseverative cognition condition, compared to the control condition. Panel B: The significant Condition × HRV interaction indicates that the association between HRV and interpersonal closeness differed between the perseverative cognition and control conditions. Specifically, the estimated HRV slopes differed significantly between conditions, with interpersonal closeness tending to decrease as HRV increased in the control condition and to increase as HRV increased in the perseverative cognition condition.

The model including Thought Distraction as a continuous predictor, Condition and Closeness Type as fixed factors, and Interpersonal Closeness ratings as the dependent variable revealed a significant Condition × Thought Distraction interaction (*χ* ^2^(1) = 4.66, *p* = 0.03), which was confirmed by a parametric bootstrap likelihood‐ratio test (*χ* ^2^(1) = 4.35, *p* = 0.047) (Figure 4, panel A). No other main effects or interactions were significant (all *ps* > 0.52). The contrast between slopes was significant (difference = 0.011, SE = 0.005, *z* = 2.16, *p* = 0.03), indicating that as thought distraction increased, interpersonal closeness decreased in the perseverative cognition induction compared to the control condition.

#### 3.3.2 Negative Emotion

The model including Negative Emotion as a continuous predictor, Condition and Closeness Type as fixed factors, and Interpersonal closeness ratings as the dependent variable did not reveal any significant main effects or interactions (all *ps* > 0.62).

#### 3.3.3 HR

The model including HR as a continuous predictor, Condition and Closeness Type and their interaction as fixed factors, and Interpersonal Closeness ratings as the dependent variable did not reveal any significant main effects or interactions (all *ps* > 0.21).

#### 3.3.4 HRV

The model including vagally mediated HRV as a continuous predictor, Condition and Closeness Type as fixed factors, and Interpersonal Closeness ratings as the dependent variable revealed a significant Condition × HRV interaction (*χ* ^2^(1) = 6.98, *p* = 0.008), which was confirmed by a parametric bootstrap likelihood‐ratio test (*χ* ^2^(1) = 6.20, *p* = 0.020) (Figure 4, panel B). No other main effects or interactions reached statistical significance (all *ps* > 0.45). Estimated marginal trends (emtrends) showed that the contrast between the slopes was significant (difference = −0.023, SE = 0.0087, *z* = 2.64, *p* = 0.008). Specifically, as HRV increased, interpersonal closeness tended to decrease in the control condition, whereas it tended to increase in the perseverative cognition condition. Thus, the association between HRV and interpersonal closeness differed significantly between the two experimental conditions. This pattern may reflect greater interpersonal engagement among participants who maintained higher parasympathetic tone despite the induction of perseverative cognition, possibly indicating a form of physiological resilience.

### 3.4 Impact of Perseverative Cognition Induction, Subjective Reports, and Physiological Responses on Automatic Imitation

#### 3.4.1 Reaction Times

RTs were analyzed using a LMM on Trial (Congruent, Incongruent, Neutral) and Condition (Perseverative Cognition vs. Control) as fixed effects, and random intercept for participants and random slope of Condition. The parametric bootstrap analysis confirmed the expected effect of Trial congruency, *χ* ^2^(2) = 71.68, *p* = 0.001. Responses were fastest in congruent trials (*M* = 431.49, SD = 76.04), slower in neutral trials (*M* = 466.66, SD = 88.48), and slowest in incongruent trials (*M* = 501.05, SD = 104.44). Neither the main effect of Condition, *χ* ^2^(1) = 1.08, *p* = 0.289, nor the Trial × Condition interaction, *χ* ^2^(2) = 0.64, *p* = 0.733, was significant.

#### 3.4.2 Facilitation

Parametric bootstrap analyses revealed no significant main effects or interactions in models including Thought Distraction, Negative Emotion, vagally mediated HRV, or HR as continuous predictors, together with Condition as a fixed factor (all *ps* ≥ 0.184).

#### 3.4.3 Interference

Parametric bootstrap analyses revealed no significant main effects or interactions in models including Thought Distraction or HR as continuous predictors, together with Condition as a fixed factor (all *ps* ≥ 0.564). Although the original asymptotic analysis indicated a significant main effect of vagally mediated HRV, this effect was not supported by the parametric bootstrap likelihood‐ratio test, *χ* ^2^(1) = 2.64, *p* = 0.105. The main effect of Condition and the Condition × HRV interaction were also not significant, *ps* = 0.370 and 0.255, respectively.

#### 3.4.4 Congruence

Parametric bootstrap analyses revealed no significant main effects or interactions in models including Negative Emotion, vagally mediated HRV, or HR as continuous predictors, together with Condition as a fixed factor (all *ps* ≥ 0.080). Although the original asymptotic analysis indicated a significant main effect of Thought Distraction, this effect was not supported by the parametric bootstrap likelihood‐ratio test, *χ* ^2^(1) = 1.46, *p* = 0.233. Neither the main effect of Condition nor the Condition × Thought Distraction interaction was significant, *ps* = 0.570 and 0.774, respectively.

## 4 Discussion

The present study investigated the impact of perseverative thinking on interpersonal engagement propensity. Specifically, we tested whether a brief induction of perseverative cognition would reduce affiliative behaviors compared to a control condition. Interpersonal engagement propensity was assessed using three indicators: perceived self‐other overlap (IOS), preferred interpersonal closeness, and automatic imitation. To identify the mechanisms underlying these effects, we also measured subjective thought‐related distraction, negative affect, HR, and vagally mediated HRV, which served as indices of the cognitive, emotional, and physiological responses to the induction of perseverative cognition. This approach allowed us to examine whether and how perseverative cognition influences interpersonal engagement propensity.

### 4.1 Perseverative Cognition Increases Thought‐Related Distraction, Negative Affect, and Sympathetic Activation

Consistent with expectations, the induction of perseverative cognition resulted in significantly higher levels of subjective distraction and negative affect compared to the control condition. Participants reported greater difficulty in disengaging from their internal thoughts and a heightened experience of negative emotions, confirming the effectiveness of the induction procedure. These results align with previous findings linking perseverative cognition to increased emotional distress and attentional narrowing in both experimental and ecological studies (Fox et al. 2015; Koster et al. 2011; reviewed in Ottaviani 2018). At the physiological level, HR was significantly elevated in the perseverative cognition condition, particularly during the first minute of the task, while HRV was reduced. This pattern supports prior research showing that even brief episodes of internally focused negative thought can trigger increased sympathetic activation and lower vagally mediated HRV (Ottaviani et al. 2016). HR gradually declined across the task, suggesting partial habituation over time.

### 4.2 Perseverative Cognition Does Not Robustly Alter Perceived Self‐Other Overlap

Although the original mixed‐effects models suggested that negative affect and HR moderated IOS ratings, these effects were not supported by the bootstrap procedure, indicating that they should be interpreted cautiously. One possible explanation is that IOS primarily reflects a relatively stable subjective representation of interpersonal connectedness that may be less sensitive to brief experimental manipulations. Alternatively, the present induction may not have been sufficiently strong or prolonged to reliably alter participants' perception of self–other overlap. Future studies employing longer or repeated inductions of perseverative cognition may help clarify whether sustained perseverative thinking influences this aspect of social cognition.

### 4.3 Perseverative Cognition Reduces Preferred Interpersonal Closeness Through Thought‐Related Distraction and Reduced Physiological Regulation

Participants reported lower preferred interpersonal closeness following the perseverative cognition induction compared to the control condition, when they experienced higher levels of thought‐related distraction. This suggests that perseverative thinking, by consuming attentional resources and increasing self‐focused cognition, reduces the cognitive and emotional resources available for interpersonal interactions. These findings are consistent with prior research suggesting that perseverative cognition interferes with social approach tendencies by narrowing attentional focus (Koster et al. 2011). Importantly, vagally mediated HRV moderated the effect of condition on closeness preferences. Participants with higher HRV reported greater closeness (i.e., a preference for reduced physical distance) in the perseverative cognition compared to the control condition, indicating that individuals with greater physiological regulatory capacity may be more resilient to the distancing effects of perseverative cognition. This aligns with the Neurovisceral Integration Model (Thayer and Lane 2009), which links HRV to the flexible engagement of prefrontal‐autonomic systems that support both emotion regulation and interpersonal behavior. Indeed, individuals with higher HRV tend to perceive the environment as safer, including social stimuli such as faces (e.g., Bagnis et al. 2023). Conversely, participants with lower HRV showed reduced closeness in the perseverative cognition condition, suggesting that lower regulatory capacity may amplify the interpersonal consequences of internally generated distress. The moderating role of HRV further underscores its importance as a resilience factor and highlights the crucial role of parasympathetic regulation in supporting adaptive behavior in the face of internal stressors.

These results suggest that attentional and physiological regulatory processes may represent more reliable pathways through which perseverative cognition influences interpersonal engagement propensity.

### 4.4 Perseverative Cognition Does Not Alter Automatic Imitation Behavior

Contrary to our expectations, automatic imitation performance did not significantly differ between the perseverative cognition and control conditions. The results showed a robust effect of trial congruency, indicating the expected interference and facilitation effects characteristic of the automatic imitation paradigm, whereas no other significant results were found. Overall, these findings suggest that a brief induction of perseverative cognition does not reliably influence automatic imitation. Future studies with larger samples will be needed to determine whether perseverative cognition exerts subtle effects on imitation control that could not be detected in the present study.

## 5 Conclusions and Limitations

Taken together, the present findings demonstrate that experimentally induced perseverative cognition can diminish interpersonal engagement propensity. Perseverative thinking was associated with increased cognitive distraction, heightened negative affect, and elevated sympathetic activation and lower vagally mediated HRV. Of these factors, only cognitive distraction and lower vagally mediated HRV predicted reduced affiliative responses, particularly reduced preferred interpersonal closeness. These results suggest that perseverative cognition impairs attentional and physiological regulation abilities essential for adapting social proximity, ultimately hindering the individual's readiness to connect with others. In future investigations, with the aim to preserve the ecology of the experimental setting and obtain more naturalist results, it might be worth studying social mimicry as a form of automatic imitation. Social mimicry involves the imitation of socially meaningful signals (e.g., facial expressions, prosody, and expressive gestures) (Seibt et al. 2015; Wang and Hamilton 2012). In conclusion, this study provides evidence of the social costs of perseverative cognition, emerging even in non‐clinical populations. By extending current knowledge of perseverative cognition into the interpersonal domain, our findings underscore the importance of investigating how it undermines social connectedness, particularly among individuals at risk for depression and anxiety.

## Author Contributions

**Martina Fusaro:** conceptualization, methodology, software, data curation, formal analysis, supervision, writing – original draft, writing – review and editing. **Vanessa Era:** conceptualization, methodology, software, data curation, formal analysis, supervision, funding acquisition, project administration, writing – original draft, writing – review and editing. **Giovanna Cuomo:** methodology, data curation, formal analysis, visualization, writing – review and editing. **Seyma Kaya:** data curation, investigation, writing – review and editing. **Cristina Ottaviani:** conceptualization, methodology, writing – review and editing. **Chiara Fini:** conceptualization, methodology, supervision, writing – review and editing.

## Funding

This work was funded by the Italian Fund for Science (Fondo Italiano per la Scienza, FIS) – FIS 2 Call, Italian Ministry of University and Research (MUR), under project FIS‐2023‐03019, CUP B53C24009640001, admitted for funding by MUR Directorial Decree No. 23178 of 10 December 2024.

## Conflicts of Interest

The authors declare no conflicts of interest.

## Supporting information


**Table S1:** Questionnaires scores.
**Table S2:** Summary of beat correction performed by Kubios HRV automatic artifact correction across all analyzed recordings.

**Data S1:** Supporting Information.

## Data Availability

The data that support the findings of this study are openly available in OSF at https://osf.io/udxhs/overview?view_only=3bf64b0db8824a91bee383b679188edb.
