# Supplementary material for: The Social Cost of Perseverative Cognition: Impairing Interpersonal Engagement Propensity Through Cognitive and Autonomic Dysregulation
Source: Psychophysiology. 2026 Aug 17;63(8):e70379. doi: 10.1111/psyp.70379 (PMC13481889; doi:10.1111/psyp.70379)
Supplement: Supplementary file 1 [file PSYP-63-e70379-s002.docx]

**Supplementary Materials**

**The social cost of perseverative cognition: impairing interpersonal engagement propensity through cognitive, emotional, and autonomic dysregulation**

Martina Fusaro^1,2^, Chiara Fini^3^, Seyma Kaya^1^, Giovanna Cuomo^1,2^?, Cristina Ottaviani^1,2^, Vanessa Era^1,2*^

¹ Department of Psychology, Sapienza University, Rome, Italy

^2^ IRCCS Fondazione Santa Lucia, Rome, Italy

^3^ Department of Psychology and Health Sciences, Pegaso Telematic University

*corresponding author: vanessa.era@uniroma1.it

**Table S1.** Questionnaires scores.

| PARTECIPANT | RRS (Treynor et al., 2003) | TRIM-18(McCullough et al., 2006) | | | PSWQ (Meyer et al., 1990) | STAI (Spielberger et al., 1983) | UCLA (Version 3; Russell, 1996) |
| --- | --- | --- | --- | --- | --- | --- | --- |
|  |  | **Avoidance** | **Revenge** | **Forgiveness** |  |  |  |
| 1 | 44 | 25 | 13 | 15 | 43 | 54 | 35 |
| 2 | 49 | 17 | 5 | 12 | 34 | 46 | 32 |
| 3 | 55 | 21 | 6 | 17 | 24 | 53 | 35 |
| 4 | 39 | 25 | 5 | 9 | 40 | 52 | 41 |
| 5 | 39 | 25 | 12 | 14 | 49 | 50 | 38 |
| 6 | 50 | 9 | 7 | 21 | 50 | 56 | 32 |
| 7 | 44 | 27 | 10 | 21 | 52 | 52 | 44 |
| 8 | 44 | 21 | 11 | 21 | 31 | 51 | 35 |
| 9 | 26 | 20 | 12 | 13 | 73 | 47 | 29 |
| 10 | 61 | 25 | 13 | 17 | 32 | 42 | 55 |
| 11 | 53 | 33 | 13 | 13 | 27 | 52 | 38 |
| 12 | 33 | 14 | 5 | 23 | 66 | 55 | 28 |
| 13 | 61 | 8 | 6 | 23 | 46 | 50 | 48 |
| 14 | 45 | 25 | 6 | 12 | 42 | 50 | 47 |
| 15 | 37 | 27 | 7 | 13 | 68 | 55 | 30 |
| 16 | 38 | 21 | 7 | 15 | 56 | 56 | 42 |
| 17 | 60 | 25 | 6 | 12 | 38 | 52 | 34 |
| 18 | 30 | 21 | 6 | 14 | 47 | 60 | 29 |
| 19 | 52 | 16 | 10 | 20 | 41 | 34 | 34 |
| 20 | 57 | 18 | 12 | 19 | 50 | 56 | 43 |
| 21 | 43 | 7 | 5 | 26 | 40 | 48 | 30 |
| 22 | 41 | 30 | 15 | 12 | 44 | 47 | 40 |
| 23 | 28 | 29 | 5 | 12 | 50 | 52 | 25 |
| 24 | 52 | 26 | 13 | 10 | 38 | 52 | 43 |
| 25 | 44 | 17 | 7 | 19 | 48 | 54 | 45 |
| 26 | 28 | 15 | 7 | 20 | 65 | 53 | 42 |
| 27 | 37 | 23 | 5 | 18 | 56 | 52 | 24 |
| 28 | 25 | 19 | 7 | 21 | 62 | 49 | 27 |
| 29 | 53 | 24 | 12 | 19 | 47 | 46 | 38 |
| 30 | 44 | 26 | 8 | 16 | 49 | 50 | 36 |
| 31 | 88 | 18 | 21 | 13 | 29 | 52 | 70 |
| 32 | 58 | 17 | 8 | 11 | 26 | 52 | 32 |
| 33 | 52 | 29 | 6 | 14 | 44 | 42 | 35 |
| 34 | 59 | 26 | 13 | 10 | 27 | 53 | 35 |
| 35 | 70 | 27 | 8 | 17 | 44 | 52 | 50 |
| 36 | 42 | 14 | 5 | 21 | 56 | 53 | 36 |
| 37 | 47 | 19 | 6 | 23 | 38 | 63 | 33 |
| 38 | 46 | 29 | 8 | 11 | 21 | 54 | 31 |
| 39 | 31 | 29 | 7 | 14 | 46 | 45 | 29 |
| 40 | 50 | 32 | 6 | 8 | 46 | 60 | 38 |
| 41 | 61 | 21 | 8 | 21 | 34 | 53 | 34 |
| 42 | 36 | 19 | 6 | 17 | 34 | 50 | 31 |
| 43 | 61 | 28 | 9 | 10 | 17 | 50 | 39 |
| 44 | 46 | 25 | 5 | 14 | 44 | 56 | 58 |
| 45 | 31 | 24 | 6 | 13 | 63 | 49 | 29 |
| 46 | 41 | 25 | 10 | 14 | 67 | 48 | 37 |
| 47 | 54 | 32 | 8 | 14 | 41 | 57 | 29 |
| 48 | 45 | 31 | 11 | 9 | 58 | 44 | 44 |
| 49 | 43 | 20 | 5 | 20 | 44 | 56 | 35 |

**Table S2. Summary of beat correction performed by Kubios HRV automatic artifact correction across all analyzed recordings.**

| Measure | Value |
| --- | --- |
| Number of ECG recordings | 547 |
| Mean corrected RR(%) | 1.52 |
| Standard deviation (%) | 2.40 |
| Median corrected RR(%) | 0.66 |
| Interquartile range (%) | 0.00–2.17 |
| Minimum (%) | 0.00 |
| Maximum (%) | 23.53 |
| Recordings with 0% corrected RR | 39.3% |
| Recordings with >5% corrected RR | 7.3% |
| Recordings with >10% corrected RR | 1.3% |
